# Patients with naproxen‐induced liver injury display T‐cell memory responses toward an oxidative (S)‐O‐desmethyl naproxen metabolite but not the acyl glucuronide

**DOI:** 10.1111/all.15830

**Published:** 2023-07-29

**Authors:** Paul Thomson, Nik Fragkas, Laila M. Kafu, Guruprasad P. Aithal, M. Isabel Lucena, Luigi Terracciano, Xiaoli Meng, Munir Pirmohamed, Dominique Brees, Gerd A. Kullak‐Ublick, Alex Odermatt, Thomas Hammond, Michael Kammüller, Dean J. Naisbitt

**Affiliations:** ^1^ Molecular& Clinical Pharmacology University of Liverpool Liverpool UK; ^2^ Novartis Institutes for BioMedical Research Basel Switzerland; ^3^ NIHR Nottingham Biomedical Research Centre and Nottingham Digestive Diseases Centre, Translational Medical Sciences, West Block, Queen's Medical Centre University of Nottingham Nottingham UK; ^4^ Unidad de Gestión Clínica de Aparato Digestivo y Servicio de Farmacología Clínica, Instituto de Investigación Biomédica de Málaga‐IBIMA, Hospital Universitario Virgen de la Victoria Universidad de Málaga, CIBERehd Malaga Spain; ^5^ Institute of Pathology University Hospital Basel Basel Switzerland; ^6^ University Hospital Zurich University of Zurich Zurich Switzerland; ^7^ Novartis Global Drug Development Basel Switzerland; ^8^ Division of Molecular & Systems Toxicology, Department of Pharmaceutical Sciences University of Basel Basel Switzerland; ^9^ Oncology Safety, Clinical Pharmacology and Safety Sciences R&D Cambridge UK

**Keywords:** drug‐induced liver injury, human, immune system, T lymphocytes

## Abstract

**Background:**

Exposure to nonsteroidal anti‐inflammatory drugs (NSAIDs) such as ibuprofen (IBU) and naproxen (NAP) is associated with idiosyncratic drug‐induced liver injury (DILI). Carboxylate bioactivation into reactive metabolites (e.g., acyl glucuronides, AG) and resulting T‐cell activation is hypothesized as causal for this adverse event. However, conclusive evidence supporting this is lacking.

**Methods:**

In this work, we identify CD4^+^ and CD8^+^ T‐cell hepatic infiltration in a biopsy from an IBU DILI patient. Lymphocyte transformation test and IFN‐γ ELIspot, conducted on peripheral blood mononuclear cells (PBMCs) of patients with NAP‐DILI, were used to explore drug‐specific T‐cell activation. T‐cell clones (TCC) were generated and tested for drug specificity, phenotype/function, and pathways of T‐cell activation. Cells were exposed to NAP, its oxidative metabolite 6‐O‐desmethyl NAP (DM‐NAP), its AG or synthesized NAP‐AG human‐serum albumin adducts (NAP‐AG adduct).

**Results:**

CD4^+^ and CD8^+^ T‐cells from patients expressing a range of different Vβ receptors were stimulated to proliferate and secrete IFN‐γ and IL‐22 when exposed to DM‐NAP, but *not* NAP, NAP‐AG or the NAP‐AG adduct. Activation of the CD4^+^ TCC was HLA‐DQ‐restricted and dependent on antigen presenting cells (APC); most TCC were activated with DM‐NAP‐pulsed APC, while fixation of APC blocked the T‐cell response. Cross‐reactivity was not observed with structurally‐related drugs.

**Conclusion:**

Our results confirm hepatic T‐cell infiltrations in NSAID‐induced DILI, and show a T‐cell memory response toward DM‐NAP indicating an immune‐mediated basis for the adverse event. Whilst bioactivation at the carboxylate group is widely hypothesized to be pathogenic for NSAID associated DILI, we found no evidence of this with NAP.

## Graphical abstract

CD4^+^ and CD8^+^ T‐cell hepatic infiltration is detected in a biopsy from a patient with NSAID (nonsteroidal anti‐inflammatory drug)‐induced liver injury. The naproxen metabolite, desmethyl naproxen, activates T‐cells from patients with naproxen‐induced liver injury. Whilst bioactivation at the carboxylate group is widely hypothesized to be pathogenic for NSAID‐induced liver injury, we found no evidence of this with naproxen.Abbreviations: HLA, human leukocyte antigen; IFN, interferon; IL, interleukin; NSAID, non‐steroidal anti‐inflammatory drug.

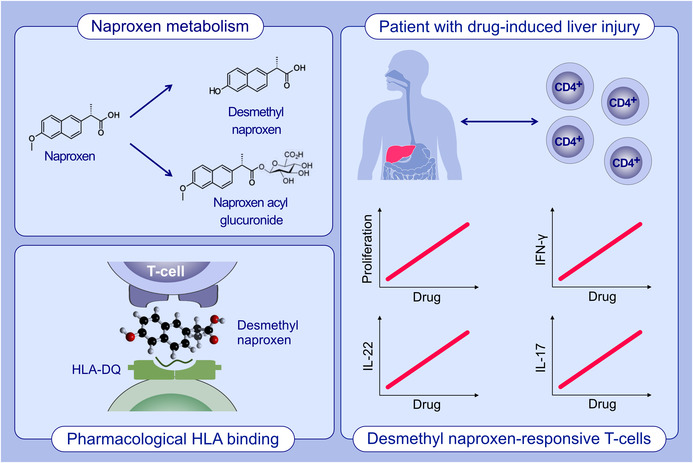

**Abbreviations**
APC: Antigen presenting cells
DILI: drug‐induced liver injury
DM‐NAP: 6‐O‐desmethyl naproxen
HLA: human leukocyte antigen
HSA: human serum albumin
IL: interleukin
NAP: naproxen
NAP‐AG: naproxen acyl glucuronide
NSAID: nonsteroidal anti‐inflammatory drug
PBMC: peripheral blood mononuclear cells
SPE: solid phase extraction
TCC: T‐cell clone
TR: Transporter negative

## 1 INTRODUCTION

Naproxen (NAP) and ibuprofen (IBU) are carboxylate nonsteroidal anti‐inflammatory drugs (NSAIDs) that are widely used for the treatment of mild to moderate pain and arthritis.^1^, ^2^, ^3^, ^4^ Human exposure to both drugs is associated drug‐induced liver injury (DILI).^5^, ^6^, ^7^, ^8^ Whilst liver reactions are rare (1–3 cases for NAP and 6–13 cases for IBU per 100,000 patient years), the clinical exposure of these compounds is vast.^9^, ^10^ Patients with liver injury report an elevation in serum aminotransferase levels over a period of 1–6 weeks after commencing therapy.

Antibody and alkaline hydrolysis liberation techniques have shown carboxylic acid drugs or their metabolic derivatives form covalent adducts with circulating and hepatic proteins.^11^, ^12^, ^13^, ^14^, ^15^, ^16^, ^17^ This established protein reactivity of carboxylate NSAIDs is hypothesized to be a critical step in eliciting the adverse reactions associated with this drug class, with the drug‐protein adduct suspected to be able to be presented to antigen presenting cells (APC) and eliciting T‐cell responses.^18^, ^19^ A common metabolic pathway of carboxylic acids is their Phase II glucuronidation forming acyl glucuronide metabolites. Whilst acyl glucuronidation is a pivotal strategy for pharmacological deactivation and clearance of carboxylate NSAIDs, they are chemically unstable in aqueous conditions degrading via hydrolysis and acyl migration.^20^, ^21^ In vitro investigations have shown acyl glucuronides to form covalent adducts with protein, forming two distinct adducts; (i) transacylation adducts through direct modification of nucleophilic amino acids and (ii) a Schiff base adduct retaining the glucuronide structure through the reaction of isomeric glucuronides with amine nucleophiles followed by Amadori rearrangement.^22^ In volunteers receiving doses of carboxylate NSAIDs, irreversible binding to circulating plasma proteins was shown to correlate with circulating acyl glucuronide exposure.^22^ This protein reactivity of acyl glucuronides has led the FDA labelling them as “toxic” in their Metabolites in Safety Testing guidance, although this notably was tempered to “potentially toxic” in the recent 2020 revision (https://www.fda.gov/media/72279/download). In accordance with this, and following Benet's demonstration of an almost perfect correlation between degradation rate of acyl glucuronide metabolites in buffer with extent of albumin binding in vitro,^23^ most pharmaceutical companies include acyl glucuronide buffer stability assessments in novel compound risk monitoring strategies.^24^ However, this concern is held in the absence of convincing evidence that acyl glucuronides or their adducts represent any immunotoxicological consequence. Furthermore, the immunogenic properties of other metabolites of carboxylate drugs have largely been unexplored.

The identification of circulating antibodies recognizing diclofenac‐modified hepatic protein in 100% of patients suffering diclofenac‐induced liver injury implicates the adaptive immune system in the pathogenesis of carboxylate NSAID liver reactions.^11^ Supporting this, a genome‐wide association study followed by high resolution HLA typing identified an association between lumiracoxib‐induced liver injury and expression of several HLA Class‐II alleles including HLA‐DRB1*15:01, ‐DQB1*06:02, and DQA1*01:02.^25^ However, to date no drug/metabolite responsive T‐cells have yet been identified in patients either currently or retrospectively suffering DILI reactions to carboxylate NSAIDs. Despite this, drug‐specific T‐cells are detected in blood of patients with hepatic adverse events associated with a number of therapeutics including flucloxacillin,^26^ amoxicillin clavulanate,^27^ atabecestat,^28^ and anti‐tuberculous drugs.^29^ In the case of flucloxacillin the drug‐derived antigen interacts with a degree of selectivity with the HLA molecule HLA‐B*57:01 identified in genome‐wide association studies.^26^, ^30^, ^31^ Effector T‐cells have been shown to infiltrate liver of patients with sulfasalazine‐,^32^ flucloxacillin‐^33^ and atabecestat‐induced liver injury,^34^ indicating that they play a direct role in the tissue damage.

In this work we used NAP and IBU as model carboxylate NSAIDs. Both NAP and IBU undergo extensive cytochrome P450 driven hepatic metabolism. NAP forming 6‐O‐desmethyl NAP (DM‐NAP) via CYP isoforms CYP1A2, CYP2C8, and CYP2C9,^20^, ^35^ and IBU forming 2‐hydroxy, 3‐hydroxy and di‐carboxy metabolites via CYP2C8 and CYP2C9.^36^ Both parent drugs and their oxidative metabolites can undergo Phase II glucuronidation,^37^ with 50% of IBU^38^ and 95% of NAP following oral doses recovered in a conjugated form in urine.^35^ The aims of this study were to (i) explore T‐cell infiltration in NSAID‐induced DILI; (ii) understand whether drug‐responsive T‐cells circulate in patients with NAP‐induced liver injury; and (iii) determine whether NAP, DM‐NAP, NAP acyl glucuronide (NAP‐AG) or NAP‐AG‐albumin adducts participate in the T‐cell response by functional and phenotyping experiments.

## 2 METHODS

### 2.1 Patient samples and in vitro experimentation

A liver biopsy from a patient suffering IBU‐induced DILI was taken, fixed in 10% formalin and embedded in paraffin. Sections were stained by haematoxylin and eosin (H&E) and prepared for immunohistochemical analysis. Standard indirect immunoperoxidase procedures were used for immunohistochemistry. Primary antibodies used were specific for CD3 (monoclonal, Dako) and CD8 (clone C8/144B, DakoCytomation).

Three patients with NAP‐induced liver injury (as defined as alanine transaminase (ALT) ≥5× upper limit of normal (ULN) or alkaline phosphatase (ALP ≥2× ULN or ALT ≥3× ULN plus total bilirubin ≥2× ULN), four NAP tolerant patients (exposed to NAP for greater than 1 year) and four individuals with no known NAP exposure were recruited to the study. Basic demographics of the cohort and adverse event details are described in Table 1. Informed consent was provided by the patients and the study was approved by the respective local Ethical Committees. A material transfer agreement was signed prior to shipment of PBMC to the University of Liverpool. A total of 60 mL of blood was collected from each donor and peripheral blood mononuclear cells (PBMC) were isolated using Lymphoprep (Axis‐shield, Dundee). PBMC were incubated with NAP, DM‐NAP, NAP‐AG (all 50–600 μM)) NAP‐AG‐albumin adduct (1 mg/mL) or tetanus toxoid (1 μg/mL)/PHA (1 μg/mL) (as positive controls) before being subjected to in vitro diagnostic testing and cloning of individual T‐cells for characterization of cellular phenotype, function, antigen specificity, and pathways of activation. Detailed methods are available as supplementary material.

**TABLE 1:** Demographics of patients and summary of diagnostic testing.

id	Gender	Age (years)	Co‐medication	Date of reaction and blood donation	Onset of symptoms (weeks)	Clinical presentation	Liver parameters			LTT / TCC		
							ALT [Ref: 2–53 IU/L]	ALP [Ref: 40–130 IU/L]	Bilirubin [Ref: 3–17 Umol/L]	NAP	DM‐NAP	NAP‐AG
DILI P1^a^	F	51	Evorel, Glucosamine	Aug 2010 July 2016	1	Severe pain and jaundice. Diagnosed with cholelithiasis, underwent cholecystectomy	596	268	47	(−/−)	**(+/+)**	(−/−)
DILI P2	F	56	Levothyroxine, Lansoprazole and Citclopram	Apr 2017 Oct 2018	2	Patient administered NAP and dihydrocodine in Jan 2017 for 5 days. Then readministered NAP on 5th April 2017. Presented with constant pain.	1286	912	105	(−/−)	**(−/+)**	(−/−)
DILI P3	F	31	Methotrexate, Folic acid, deflazacort, omeprazole	Apr 2011 Mar 2018	3	Severe pain and jaundice.	535	146	16.9	(−/−)	(−/−)	(−/−)
DILI PIBU1^b^	M	52	Novalgin	Dec 2021	1	Mild abdominal pain and jaundice	1632	272	308	na	na	na
TP 1^c^	F	64	Omeprazole, Ibuprofen, Co‐codamol	na	na	na	na	na	na	(−/−)	(−/−)	(−/−)
TP 2	M	65	Sildenopril, Ambrosentin, Qinine, Aspirin, Levothyroxine, Omeprazole, Venlaflaxine	na	na	na	na	na	na	(−/−)	(−/−)	(−/−)
TP 3	F	45	Febuxostat	na	na	na	na	na	na	(−/−)	(−/−)	(−/−)
TP 4	F	64	Methotrexate, TNFa, Omeprazole, Folic Acid, Sulfasalazine	na	na	na	na	na	na	(−/−)	(−/−)	(−/−)
HD 1^d^	M	28	Na	na	na	na	na	na	na	(−/−)	(−/−)	(−/−)
HD 2	F	24	na	na	na	na	na	na	na	(−/−)	(−/−)	(−/−)
HD 3	F	59	na	na	na	na	na	na	na	(−/−)	(−/−)	(−/−)
HD 4	M	27	na	na	na	na	na	na	na	(−/−)	(−/−)	(−/−)

*Note*: The bold + indicates drug‐responsive memory T‐cells were successfully characterized by way of LTT or by generating drug responsive T‐cells. Conversely the bold − indicates circulating drug‐responsive T‐cells were not detected by way of LTT or generation of drug responsive T‐cell clones.

Abbreviations: n/a, not applicable.

^a^ NAP DILI patients

^b^ IBU DILI patient

^c^ NAP tolerant patients

^d^ drug‐naïve healthy donors.

## 3 RESULTS

### 3.1 Histopathological analysis of a liver biopsy taken from an IBU DILI patient at the time of the adverse event

**FIGURE 1:**
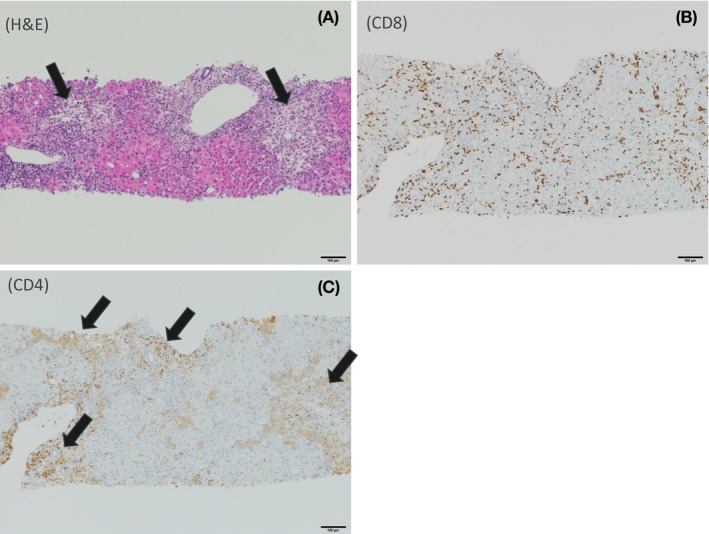
Liver biopsy from a IBU DILI patient reveals hepatic T‐cell infiltration and co‐localization with hepatic damage. (A) Liver parenchyma with diffuse confluent portal as well as porto‐central bridging necrosis (black arrows). Portal tracts appear to be less involved (H&E staining, 100× magnification). (B) Severe CD8^+^ lymphocyte infiltration (brown immunohistochemical staining), mainly located in areas of central confluent necrosis (100× magnification). (C) CD4 lymphocyte infiltration (brown immunohistochemical staining) mainly identified in portal tracts (black arrows, 100× magnification).

Liver biopsy revealed portal and lobular hepatitis with architectural distortion due to massive portoportal as well as porto‐central bridging liver cell necrosis with “passive” fibrous septa of at least 60% of the liver tissue (Figure 1). The viable hepatocytes exhibited degenerative changes with ballooning, without visible lipid accumulation. Focally, intracanalicular bilirubinostasis and some apoptotic bodies were readily identified through the lobule. In addition, an increase in eosinophilic granulocytes and plasma cells was present in portal tracts. No signs of a preexisting liver disease were seen. Portal tracts displayed mild fibrosis. Lobular infiltration by CD8^+^ lymphocytes mainly in the areas of confluent necrosis was detected. CD4^+^ lymphocytes were mainly located in portal tracts.

### 3.2 Stability of naproxen acyl glucuronide

**FIGURE 2:**
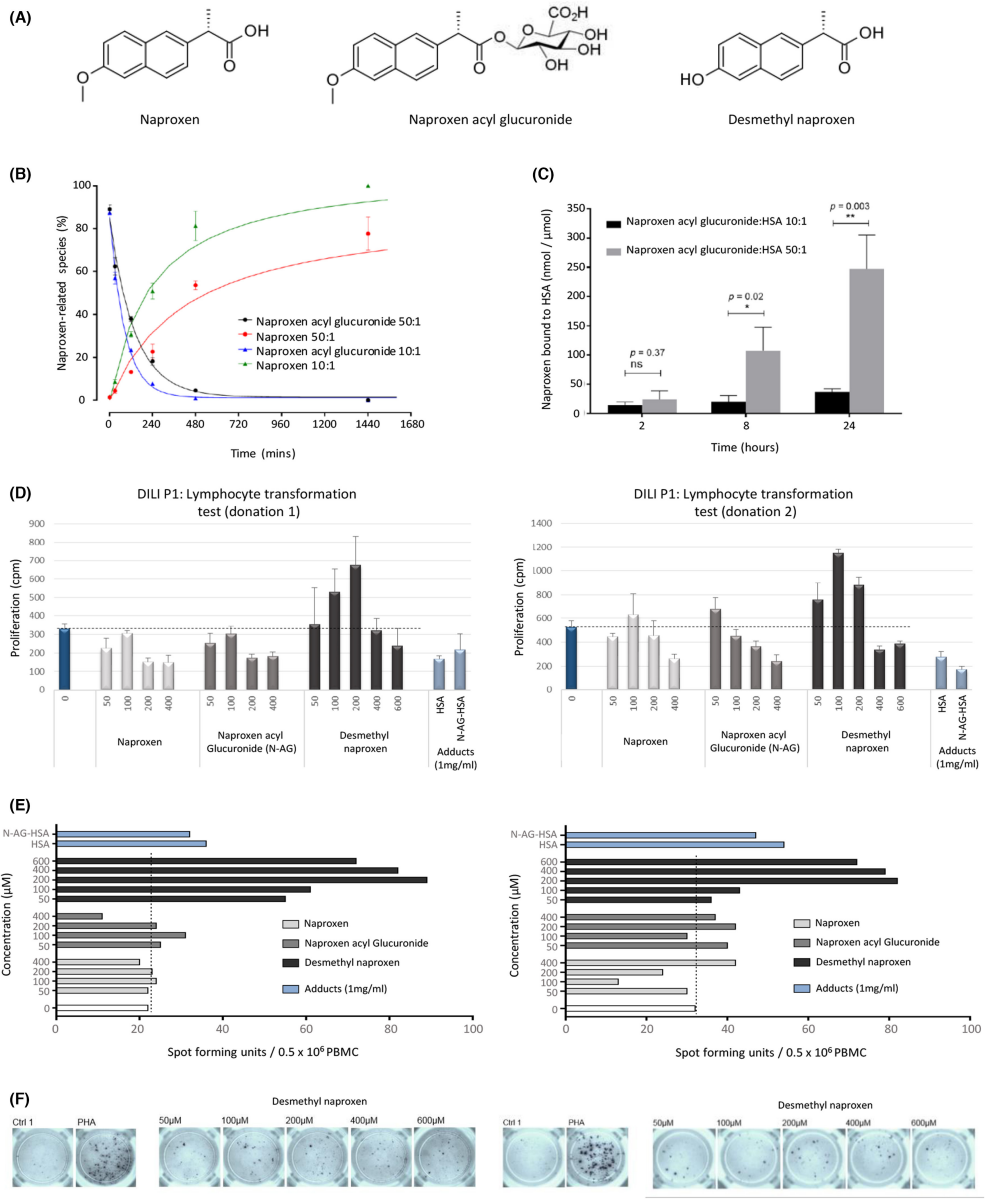
Stability and albumin binding of NAP‐AG and activation of patient T‐cells with NAP and its metabolites. (A) Structures of NAP, DM‐NAP and NAP‐AG. (B) Fitted regression curves presenting the degradation and hydrolysis of NAP‐AG during incubation with albumin (20 μM) at 1 mM (50:1) or 200 μM (10:1) in pH 7.4 at 37°C. All incubations were carried out in triplicate. Data are presented as mean ± SD of % NAP‐AG remaining or NAP formed. Data points representing the degradation of NAP‐AG are fitted with exponential decay equation (50:1: *r* ^2^ = 0.9859, 10:1; *r* ^2^ = 0.9951) while data points representing formation of NAP are fitted with hyperbola equation (50:1; *r* ^2^ = 0.9372, 10:1; *r* ^2^ = 0.9741). (C) Irreversible binding of NAP‐AG (1 mM or 200 μM) incubated with albumin (20 μM) (pH 7.4) at 37°C. Binding was measured with SPE/alkaline hydrolysis method at 2, 8 and 24 h. All incubations were performed in triplicate. Data are presented as mean ± SD of drug metabolite bound to albumin. T‐tests were used for comparisons at the same time points. (D) Lymphocytes from NAP DILI P1 were incubated with NAP, DM‐NAP, NAP‐AG and the NAP‐AG albumin conjugate for a period of 5 days in a 96 well U‐bottomed plate (37°C; 5% CO_2_). Culture medium was used as a negative control. [^3^H]thymidine was added for the final 16 h of the experiment and proliferation was assessed by scintillation counting. Bars denote mean of triplicate wells. Error bars denote ± SEM. (E) Lymphocytes from NAP DILI P1 were incubated in an ELIspot plate pre‐coated for IFN‐γ with NAP, DM‐NAP, NAP‐AG, and the NAP‐AG albumin conjugate a period of 2 days. PHA (10 μg/mL) and medium were used as positive and negative controls, respectively. The ELIspot plates were developed following the manufacturer's instructions and counted using ELIspot AID reader. (F) ELIspot images from DM‐NAP‐treated wells.

The half‐life of synthetic NAP‐AG degradation in phosphate buffer (Figure S1) and HSA solution (50–1 and 10–1 AG‐HSA Molar ratios, Figure 2B) as estimated by nonlinear regression analysis of the first‐order rates of loss of the AG isoform was 163.30, 96.45, and 58.34 min, respectively (parameters described in Table S1). The 163.30 min half‐life of NAP‐AG in phosphate buffer falls within the category of other acyl glucuronide forming compounds holding “warnings” or “withdrawn” due to their potential to elicit immune‐mediated adverse reactions,^39^ and is in alignment with previous studies investigating NAP‐AG.^39^, ^40^

Hydrolysis was found to represent an important degradation route for NAP‐AG in the presence of HSA. Increased rate and extent of hydrolysis was observed in the 10–1 compared to 50–1 (AG:HSA molar ratio) conditions (Figure 2B).

### 3.3 Irreversible binding of naproxen acyl glucuronide to albumin

NAP‐AG irreversibly bound to HSA in a concentration and time‐dependent manner (Figure 2C). Significantly increased NAP‐AG binding was observed for the 50:1 than 10:1 (NAP‐AG:HSA molar ratio) incubations at 8 and 24 h timepoints. Irreversible binding of NAP‐AG increased throughout the time‐course of incubation. Hence, maximal irreversible binding characterized was observed for 50:1 incubation at the 24 h timepoint. Adducted HSA for cell culture experiments was generated via incubation of Nap‐AG with HSA (50:1 AG:HSA molar ratio) for 24 h. Irreversible bound drug was characterized was equivalent to those derived in the kinetic studies (mean bound NAP 241.35 ± 84.77 nmol/μmol HSA), with only a minor and nonsignificant loss of bound NAP following incubation of the adducted HSA in cell culture media for 16 h (NAP 182.90 ± 87.32 nmol/μmol HSA).

### 3.4 Lymphocyte Transformation Test and PBMC ELIspot

Two separate blood donations were obtained from DILI P1, while only one was obtained from DILI P2 and P3. PBMC from DILI P1 were stimulated to proliferate weakly (Figure 2D) and secrete IFN‐γ (Figure 2E, F) in response to treatment with DM‐NAP. A decrease in the proliferative response with high DM‐NAP concentrations may relate to direct PBMC toxicity (approximately 30% and 50%, at 400 and 600 μM, respectively). In contrast, PBMC did not proliferate or secrete IFN‐γ when co‐incubated with NAP, NAP‐AG, or the NAP‐AG‐albumin conjugate. Very low levels of proliferation (stimulation index [proliferation in test cultures with drug/proliferation in control cultures] = 2) was observed when PBMC from DILI P2 were incubated with a single concentrations of DM‐NAP (600 μM), (100 μM) and NAP‐AG (200 μM) (Figure S2); however, given the low cpm recorded in medium control wells, a lack of dose‐dependency in the response and the failure to detect IFN‐γ secretion, the data should be regarded with caution. PBMC from DILI P3, all four long‐term exposed NAP tolerant patients (TP 1–4) and naïve healthy controls (HC 1–4) did not proliferate or secrete IFN‐γ in the presence of the test compounds (stimulation index <1.5) (data summarized in Table 1). Drug (metabolite) structures are shown in Figure 2A.

### 3.5 Generation of desmethyl naproxen‐responsive TCC

**FIGURE 3:**
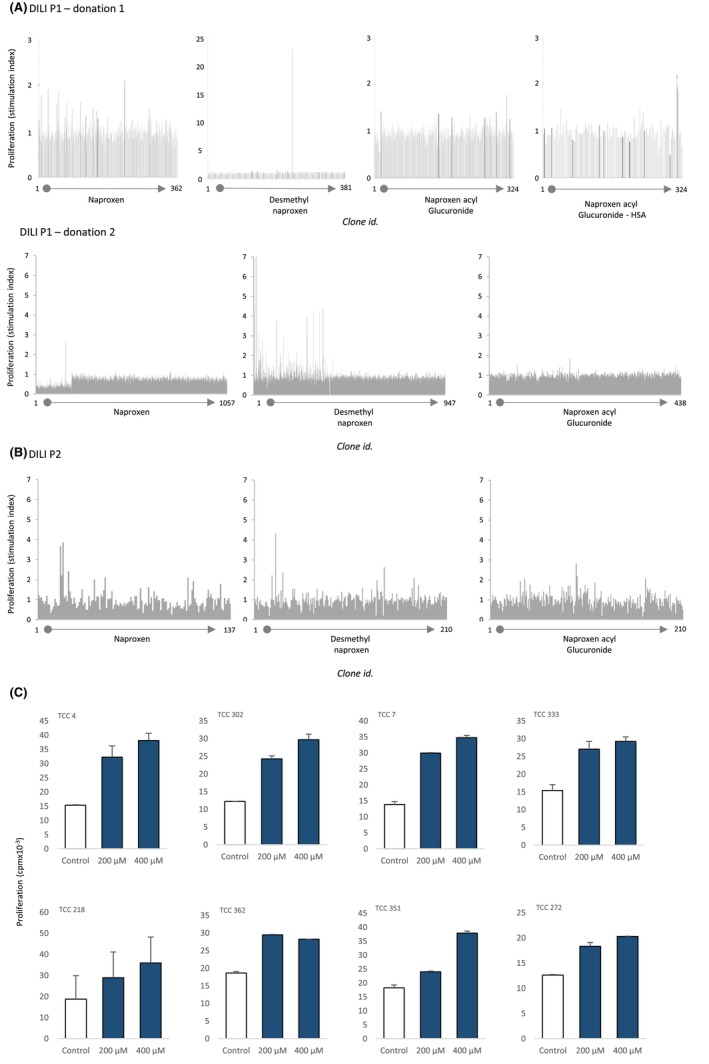
Initial drug specificity testing of TCC from patients with NAP‐induced liver injury. (A) TCC (5 × 10^4^/50 μL) were incubated with autologous EBV‐transformed B cells (1 × 10^4^/50 μL) in the presence and absence of NAP, DM‐NAP NAP‐AG or NAP‐AG albumin conjugate in a U‐bottomed 96 well microplate. Cells were incubated for 48 h (37°C; 5% CO_2_) and [^3^H]thymidine was added for the last 16 h. Proliferative responses were assessed using scintillation counting. TCC with a stimulation index of 1.5 were selected, expanded and subjected to dose–response studies. (B). Dose‐dependent activation of TCC with DM‐NAP. (C) TCC were incubated with autologous EBV‐transformed B cells and DM‐NAP (200–400 μM) and proliferative responses were measured as described above. Responses from eight representative TCC are shown.

To further investigate DILI patient T‐cell responses to NAP and its metabolites, TCC were generated from NAP, DM‐NAP, NAP‐AG, and NAP‐AG‐albumin conjugate T‐cell lines and tested for drug specificity. A total of 19 DM‐NAP‐responsive TCC were detected on initial testing (DM‐NAP 200 μM, duplicate cultures) from a total of 1328 DILI P1 TCC tested (Figure 3A). Twelve of these TCC displayed dose‐dependent proliferative responses in the presence of DM‐NAP and were expanded for more detailed mechanistic investigations (eight of the TCC are shown in Figure 3C). Far fewer DILI P1 TCC displayed proliferative responses to NAP, NAP‐AG, or the NAP‐AG‐albumin adduct and none of these were deemed drug‐responsive in dose–response studies. From a total of 210 TCC generated from DILI P2 DM‐NAP T‐cell lines, one was stimulated to proliferate in the presence of DM‐NAP on initial testing and repeat dose–response studies (Figure 3B). A small number of TCC from NAP‐ and NAP‐AG‐treated PBMC displayed stimulation index values of approximately two, on initial testing (when medium and a single drug concentration are tested). These clones were expanded and assayed in dose–response studies. Unlike the DM‐NAP TCC, clones from NAP‐ and NAP‐AG‐treated PBMC did not proliferate (results not shown) and hence the initial borderline responses were clearly false positive. Drug‐responsive TCC were not detected from DILI P3.

### 3.6 Phenotype and cytokine secretion from desmethyl naproxen specific TCC

**FIGURE 4:**
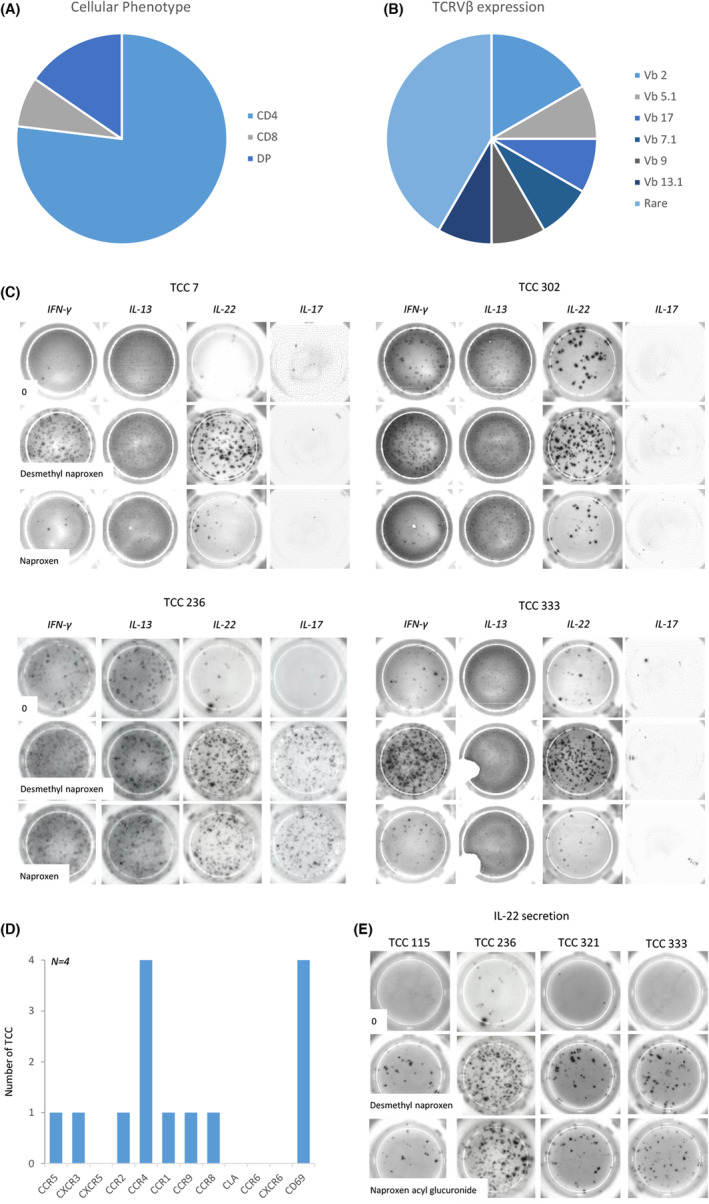
Characterization of DM‐NAP‐responsive TCC surface phenotype and cytokine secretion profiles with NAP, DM‐NAP and NAP‐AG. (A) CD4/8 phenotype of TCC. T‐cells (5 × 10^4^; 50 μL) were stained with antibodies CD4‐FITC/APC and CD8‐PE and incubated for 20 min at 4°C. TCC were then washed and analyzed using flow cytometry (BD FACSCANTO II). DP (double positive) TCC expressed high levels of CD4 and CD8. (B) TCC TCRVβ expression. T‐cells (5 × 10^4^/50 μL) were stained with T‐cell Vβ receptor antibodies (FITC, PE, FITC‐PE) and incubated for 20 min. TCC were then washed and analysed for TCRVβ expression using flow cytometry. Rare refers to TCC where Vβ expression was not detected using a panel of antibodies that covers 80% of known T‐cell receptors. (C) T‐cells (5 × 10^4^/50 μL) were incubated with autologous EBV‐transformed B cells (1 × 10^4^ / 50 μL) in an ELIspot plate, pre‐coated for IFN‐γ, IL‐13, IL‐17 or IL‐22, in the presence of NAP or DM‐NAP (400 μM) for a period of 48 h (37°C; 5% CO_2_). The ELIspot plates were developed and counted using an AID ELIspot reader. (D) TCC (50 μL) were stained with antibodies CCR2, CXCR3, CCR1, CCR8, CCR9, CTLA4, CLA, CCR6, CXCR6, CD69, CXCR5, CCR5, E‐cadherin and CCR4. TCC were then washed and analysed for receptor expression using flow cytometry. Receptor was deemed to be expressed if mean staining intensity exceeded twice the isotype control. (E) Reactivity of DM‐NAP‐responsive TCC toward NAP‐AG. IL‐22 ELIspot was used as a measure of TCC activation.

Ten of the TCC generated to DM‐NAP were phenotyped as CD4^+^, while two expressed a CD8^+^ phenotype (Figure 4A). The TCC expressed a varied TCR‐Vβ repertoire with 60% of the TCR‐Vβs detected by the commercial staining kit (Figure 4B). DM‐NAP‐responsive TCC exhibited strong IFN‐γ and IL‐22 secretion in response to incubation with the drug metabolite (Figure 4C). IL‐17 secretion was detected from one DM‐NAP‐responsive TCC. IL‐5, granzyme B, perforin, and FasL were not secreted from DM‐NAP stimulated clones. Four representative DM‐NAP‐responsive TCC were selected for cell surface receptor analysis. Across these four TCC a high expression of CCR4 and CD69 was observed, while CCR5, CXCR3, CCR2, CCR1, CCR9, and CCR8 expression was identified on one TCC (Figure 4D).

### 3.7 Desmethyl naproxen‐responsive TCC display reactivity against naproxen acyl glucuronide, but only weak reactivity against the parent drug

Very little cross‐reactivity was observed when the DM‐NAP‐responsive TCC were cultured with NAP; however, one TCC (TCC 236) was found to secrete IFN‐γ, IL‐17, and IL‐22 in the presence of both DM‐NAP and the parent drug (Figure 4C). Based on the TCC cytokine secretion profiles, IL‐22 secretion was selected as the readout to assess NAP‐AG cross‐reactivity and the pathway of drug presentation to the TCC. Interestingly, the DM‐NAP‐responsive TCCs (including TCC 236) were stimulated to secrete IL‐22 in the presence of NAP‐AG. Figure 4E shows the IL‐22 ELIspot data from 4 representative TCC.

**FIGURE 5:**
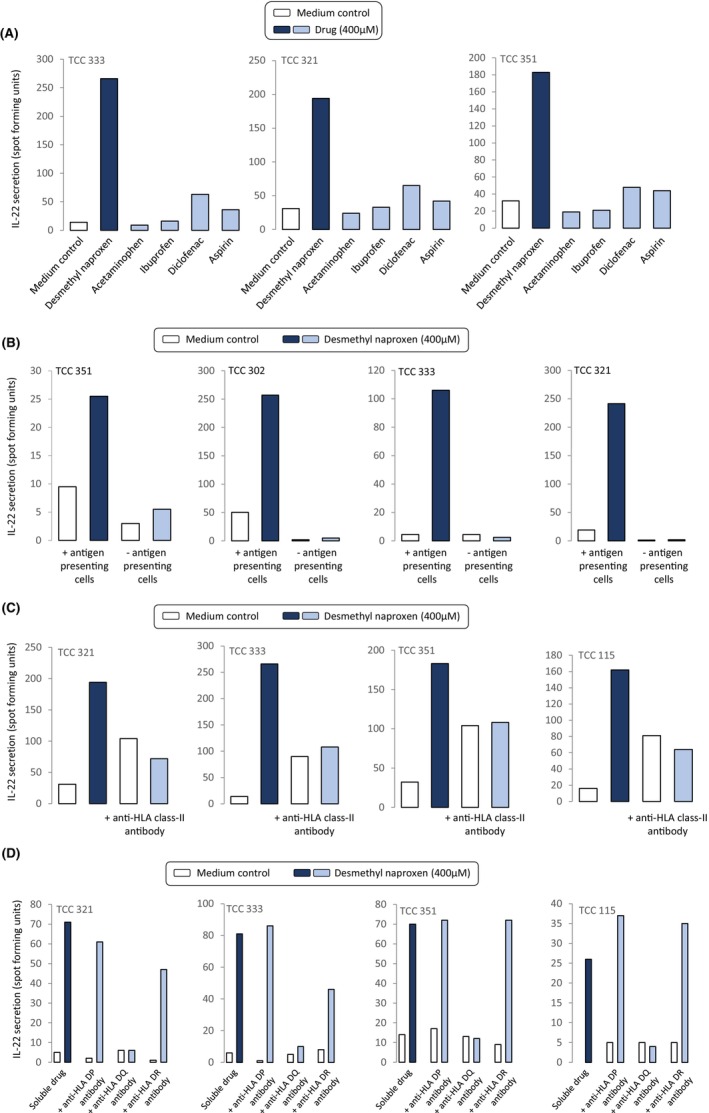
HLA‐DQ restricted activation of DM‐NAP‐responsive TCC and with other NSAIDs. (A) TCC (5 × 10^4^ / 50 μL) were incubated with autologous EBV‐transformed B cells (1 × 10^4^/50 μL) in the presence and absence of NSAID drugs in a U‐bottomed 96 well microplate. Cells were incubated for 48 h (37°C; 5% CO_2_). IL‐22 secretion was measured using ELIspot. (B) TCC (5 × 10^4^ / 50 μL) were incubated DM‐NAP in the presence or absence of autologous EBV transformed APC (1 × 10^4^/50 μL) on an ELIspot plate pre‐coated with IL‐22 for 48 h (37°C; 5% CO_2_). (C, D) TCC (5 × 10^4^ / 50 μL) were incubated with DM‐NAP and autologous EBV transformed APC (1 × 10^4^ / 50 μL) in the presence and absence of antihuman HLA blocking antibodies; (C) HLA class‐I and HLA class‐II antibodies and (D) HLA class‐II sub‐class (HLA‐DP, DQ and DR). EBV‐transformed B cells were pre‐treated with antibodies for 20 min. TCC activation was quantified via analysis of IL‐22 secretion using ELIspot.

DM‐NAP‐responsive TCC were not stimulated to secrete IL‐22 in the presence of other NSAIDs (IBU, diclofenac, aspirin, and acetaminophen) (Figure 5A).

### 3.8 HLA‐DQ‐restricted activation of desmethyl naproxen‐responsive TCC

Incubation of DM‐NAP‐responsive TCC with the drug metabolite in the presence and absence of EBV‐transformed B‐cells indicated a complete eradication of IL‐22 secretion when APC were removed from the assay (Figure 5B). Next, antihuman HLA blocking antibodies were used to explore the dependence of MHC on the activation of CD4^+^ TCC. IL‐22 secretion was detected from all TCC cultured with DM‐NAP and EBV‐transformed B‐cells (Figure 5C); however, IL‐22 secretion was reduced to basal levels when the APC were pre‐treated with an anti‐HLA Class‐II blocking antibody. Such findings were not observed in T‐cells co‐incubated with EBV transformed B‐cells pre‐treated with anti‐HLA Class‐I block. In similar antibody blocking experiments with those specific for individual MHC Class II alleles, activation of the TCC with DM‐NAP was found to be dependent on HLA‐DQ (Figure 5D). A slight reduction in the levels of IL‐22 secretion from two clones was observed with DR block; this likely relates to a nonspecific effect of adding the antibody to the assay or experimental variation with no biological significance.

### 3.9 Desmethyl naproxen‐responsive TCC are activated via a hapten mechanism

**FIGURE 6:**
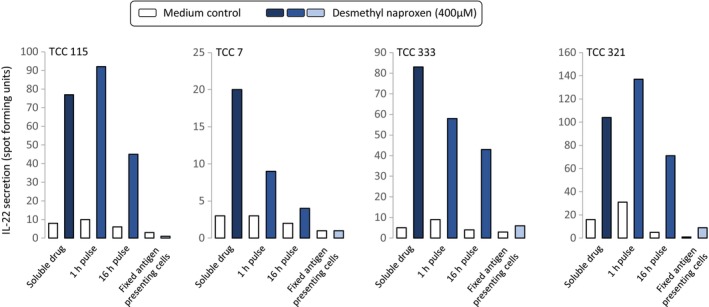
Activation of TCC with DM‐NAP‐pulsed APC is dependent on antigen processing. (A) TCC (5 × 10^4^ / 50 μL) were incubated with autologous EBV‐transformed B cells (1 × 10^4^ / 50 μL) pulsed with DM‐NAP (400 μM) for 1 or 16 h. The DM‐NAP‐pulsed EBV‐transformed B cells were washed repeatedly to remove free compound prior to culturing with T‐cells. Furthermore, TCC were incubated with DM‐NAP (400 μM) in the presence of gluturaldehyde‐fixed autologous EBV‐transformed B cells. Soluble drug was used as a positive control. Activation of the TCC was measured via IL‐22 ELIspot.

EBV‐transformed B‐cell pulsing experiments were conducted to investigate the nature of the DM‐NAP HLA‐DQ binding interaction involved in activation of the TCC. In these experiments EBV‐transformed B‐cells were cultured in the presence and absence of DM‐NAP for 1 and 16 h. The APC were subjected to repeated washing before addition to TCC in the absence of soluble drug. Figure 6 shows that most TCC secreted IL‐22 in the presence of EBV‐transformed B‐cells pulsed with DM‐NAP for 1 and 16 h and that the levels of IL‐22 secreted were similar to that observed with the soluble drug metabolite. In contrast, IL‐22 secretion was not detected when TCC were cultured with DM‐NAP and glutaraldehyde‐fixed EBV‐transformed B‐cells, where fixation blocks antigen processing.^41^

One DM‐NAP‐responsive TCC that proliferated weakly in response to DM‐NAP displayed a contrasting T‐cell activation pathway. This TCC was stimulated to proliferate in the presence of fixed EBV‐transformed B‐cells, while proliferation was not detected with APC pulsed with DM‐NAP for 16 h (Figure S3).

## 4 DISCUSSION

Direct glucuronidation of carboxylate compounds into acyl glucuronide metabolites continues to represent a challenge in the development of new pharmaceuticals. Almost four decades ago, acyl glucuronides were identified to exhibit intrinsic chemical instability and protein reactivity,^42^, ^43^ leading to hypotheses of these metabolites representing immunogenic haptens, playing a critical causal step in the pathogenesis of the hepatotoxicity associated with their parent carboxylate drugs.^43^, ^44^, ^45^ Accordingly, pharmaceutical companies incorporate assessments of acyl glucuronide risk in novel carboxylate compound development.^24^, ^46^, ^47^ Despite intensive study over the past four decades no evidence has yet been presented convincingly revealing acyl glucuronides to generate immunogenic epitopes. To our knowledge, this study represents the first detailed immunological characterization of patients suffering a delayed adverse reaction to a carboxylic acid drug, with the responsive patients showing an immunological memory to the oxidative DM‐NAP metabolite, but not NAP‐AG. A recent case‐study further supporting the association of adaptive immunity and T‐cells in delayed DILI reactions is atabecestat (a BACE inhibitor developed for the treatment of Alzheimer's disease, but terminated due to liver enzyme elevations in some patients^48^). Hepatic T‐cell infiltrates are observed in patients suffering atabecestat‐induced liver injury, while immunophenotyping work revealed T‐cells in the circulation reactive toward a primary atabecestat metabolite.^28^, ^34^

In this study we characterized the immunological basis of adverse reactions associated with carboxylic acid drugs. Whilst NAP was used as a model compound for immunophenotyping work, we could not find biopsies from any patients with NAP‐induced liver injury. However, a liver biopsy taken from an IBU‐DILI patient revealed hepatic infiltration of CD4^+^ and CD8^+^ T‐cells, supporting the pathogenic role of T‐cells in carboxylate drug‐induced liver reactions. PBMC stimulation assays and T‐cell cloning were used to identify whether patients previously suffering NAP‐induced liver injury exhibited an immunological memory toward NAP or its major metabolites, and subsequently provide detailed mechanistic characterization of these responses. In DILI P1 low levels of PBMC proliferation and IFN‐γ secretion were observed in response to DM‐NAP. In contrast, activation of PBMC was not identified with NAP or NAP‐AG. T‐cell cloning allowed identification of TCC with confirmed responsive toward DM‐NAP in two thirds of patients studied. However, again, TCC responsive toward NAP or NAP‐AG were not generated. It should be noted that DILI reactions are diagnosed based on weight of evidence and exclusion criteria, namely raised ALTs when not attributale to other factors^49^, thereby this may not represent a true case of DILI. Conversely, it is possible that circulating drug‐responsive T‐cell titers had dropped to levels below detection in the third patient. Given their was 7 years between development of the adverse event and blood sampling for immunological investigations. For severe skin reactions there is some literature evidence to suggest that the time between an adverse event and blood sampling impacts on the detection of drug‐specific T‐cells;^50^ however, in DRESS, less severe skin reactions and drug‐induced liver injury we and others have detected T‐cell responses in historic patients many years after a reaction. However, it is also possible that the precursor frequency of T‐cells in the patients' blood was below the limit or detection, or the reaction was initiated via a different mechanism or even a different drug. The in vivo *C*_max_ for NAP has been measured at 199 μM,^45^ a nontoxic concentration for PBMC, and as such initial TCC testing and most mechanistic studies were performed using a concentration of 200 μM NAP and NAP metabolites. DM‐NAP‐responsive TCC were activated in a dose‐dependent manner, with metabolite concentrations as low as 25 μM stimulating proliferative responses and cytokine release (results not shown). Twenty‐five μM is approximately 10‐fold higher than the estimated plasma *C*_max_ of DM‐NAP; however, it is possible that DM‐NAP may accumulate to significantly higher concentrations in liver due to the expression of drug metabolism enzymes and transporter proteins.

It could be argued that the chemical instability of NAP‐AG could lead to reduced compound exposure, but to mitigate this, in PBMC stimulation experiments, fresh NAP‐AG was added every 8 h. Furthermore, as a secondary approach to mitigate potential NAP‐AG degradation leading to reduced compound exposure, a NAP‐AG albumin conjugate was synthesized and added directly to the lymphocyte transformation test and PBMC ELIspot assays. Albumin was selected as the carrier protein as (i) previous studies exploring β‐lactam immunogenicity have shown that irreversibly‐modified β‐lactam albumin adducts activate T‐cells from hypersensitive patients,^51^, ^52^, ^53^ and (ii) there is a detailed understanding of the interactions between acyl glucuronides and albumin.^22^ However, again no T‐cell responses were observed to the NAP‐AG albumin conjugate

To totally rule out the presence of NAP‐AG responsive TCC, all clones primed to NAP‐AG reaching a proliferation stimulation index of approximately 1.5 were expanded and tested for NAP‐AG responsiveness. None showed any response to NAP‐AG, confirming these TCC not to be NAP‐AG responsive. Consequently, the identification of DM‐NAP and failure of NAP‐AG to generate drug‐responsive TCC supports the premise that DM‐NAP is the critical immunogenic epitope driving T‐cell responses experienced by the patients. Detection of T‐cells that are predominantly activated by a primary drug metabolite is rare, but not unprecedented. Allopurinol hypersensitivity is mediated by the dose‐dependent activation of oxypurinol‐specific T‐cells.^54^, ^55^, ^56^ Oxypurinol is a metabolite formed through the xanthine oxidase‐catalysed metabolism of allopurinol. Similarly, T‐cells from patients hypersensitive to carbamazepine and atabecestat are activated with stable metabolites alongside the parent drug.^31^, ^57^, ^58^ These data clearly highlight the importance of studying different forms of a culprit drug in in vitro diagnostic assays, especially when the parent compound yields negative results.

In agreement with the IBU DILI biopsy assessment showing CD4^+^ and CD8^+^ infiltrates, DM‐NAP was found to activate CD4^+^ and CD8^+^ T‐cells. The majority of DM‐NAP responsive TCC exhibited a CD4^+^ phenotype, but expressed different TCR‐Vβ surface receptors. This indicates these T‐cells to have originated from different precursors. TCC were stimulated to proliferate and secrete cytokines such as IFN‐γ and IL‐22 when cultured with APC and DM‐NAP. No cross‐reactivity of TCC was identified toward acetaminophen or other carboxylate NSAIDs, indicating the specificity of the responses observed, and demonstrating that these responses are unlikely attributable to a simple pharmacologic inhibition of the cyclooxygenase enzyme. This supports the hypothesis of a specific drug‐MHC‐T‐cell interaction. DM‐NAP CD4^+^ TCC did exhibit cross‐reactivity to NAP‐AG and to a lesser extent the parent drug. This could indicate that TCC exhibit some epitope recognition at sites conserved between DM‐NAP and NAP‐AG. We do not believe this cross‐reactivity to be indicative of NAP or NAP‐AG driving T‐cell sensitization as both compounds failed to generate PBMC responses or responsive TCC in any DILI patient.

Mechanistic studies (i) omitting APC from the assays and (ii) adding antihuman HLA blocking antibodies, revealed the importance of HLA‐mediated drug presentation in the pathogenesis of DM‐NAP driven T‐cell responses. Specifically, T‐cells were activated when the drug metabolite associated with HLA‐DQ molecules. The pathway of DM‐NAP presentation to T‐cells was studied using APC pulsing and fixation experiments. APC pulsed for 1 or 16 h with DM‐NAP activated the majority of TCC, while fixation blocked T‐cell activation. Collectively, these data suggest that for T‐cell activation DM‐NAP associates strongly with APC and antigen processing is a prerequisite; however, additional structural studies are needed to define the precise nature of the DM‐NAP‐HLA‐DQ peptide binding interaction. Notably, one TCC exhibited distinct characteristics from the rest. This TCC secreted IL‐17 in conjugation with IFN‐γ and IL‐22. Furthermore, the TCC was not activated in the presence of DM‐NAP pulsed APC, while fixation of the APC did not inhibit the response indicating utilization of the pharmacological interaction model of reactivity by this TCC.^59^ These data provide evidence for a heterologous population of DM‐NAP T‐cells being detected, that display different requirements for drug metabolite HLA binding in the same patient.

Collectively our findings demonstrate that T‐cells with specificity toward DM‐NAP circulate in patients with NAP‐induced liver injury. It is therefore likely that the hepatic reactions experienced by patients involve the adaptive immune system. Despite intensive investigations, we found no evidence that NAP‐AG activates patient T‐cells. Speculatively this may suggest oxidative metabolites could be causative of the ADRs in these patients.

## AUTHOR CONTRIBUTIONS

PJT, NK, LMK and XM conducted the biological experiments. GPA, ML, LT, and MP designed the clinical protocols and collected the patient samples. DJN, MK, TH, AO and GAK‐U designed the research study and supervised the project. PJT, NK, LK, TH and DJN analysed the data and drafted the manuscript. All authors critically reviewed the manuscript.

## FUNDING INFORMATION

This project received financial support from Novartis and from the MRC in the form of the MRC Centre for Drug Safety Science (grant number G0700654) and a project grant (grant number MR/R009635/1).

## CONFLICT OF INTEREST STATEMENT

DB, GK‐U and MK are employees of Novartis. PT is an employee of AstraZeneca. TH is an employee and shareholder of AstraZeneca. MP has received partnership funding for the following: MRC Clinical Pharmacology Training Scheme (co‐funded by MRC and Roche, UCB, Eli Lilly and Novartis). MP has developed an HLA genotyping panel with MC Diagnostics, but does not benefit financially from this. Both MP and DN are part of the IMI Consortium ARDAT (www.ardat.org).

## Supporting information


Data S1:

Figure S1.

Table S1.

## Data Availability

The data that support the findings of this study are available from the corresponding author upon reasonable request.
