# Supplementary material for: Patients with naproxen‐induced liver injury display T‐cell memory responses toward an oxidative (S)‐O‐desmethyl naproxen metabolite but not the acyl glucuronide
Source: Allergy. 2023 Jul 29;79(1):200–14. doi: 10.1111/all.15830 (PMC10952231; doi:10.1111/all.15830)
Supplement: Supplementary file 1 — Data S1: [file ALL-79-200-s002.docx]

**Supplemental Methods**

**Compound preparation for naproxen and naproxen metabolite assays**

NAP, DM-NAP and NAP-AG were dissolved in culture medium at a stock concentration of 1 mg/ml (structures shown in Figure 1). LC-MS loading buffer consisted of 10% acetonitrile (v/v) containing 1% lactic acid (v/v). Culture medium consisted of RPMI-1640 supplemented with pooled heat-inactivated human AB serum (10%, v/v), HEPES (25 mM), L-glutamine (2 mM), transferrin (25 μg/mL), streptomycin (100 μg/mL), and penicillin (100 U/mL). For preparation of NAP-AG adducted human serum albumin (HSA) for immunological studies 2.5 mM NAP-AG was incubated with 50 μM HSA in 0.1 M phosphate buffer (pH 7.4) at 37°C for 24 hours. Lactic acid (1 % v/v) was added to prevent further NAP-AG protein binding. HSA was separated from unbound NAP-AG by size exclusion chromatography (see supplemental methods) for use in T-cell studies. Non-adducted HSA was subjected to the same procedure was used as a negative control. Aliquots of adducted and non-adducted HSA were stored at -80 ^o^C following preparation and thawed immediately before use. Purity and stability of the NAP-AG-HSA conjugate was assessed after 0 and 16 hours in culture medium by LC-MS.

## **Naproxen acyl glucuronide stability and protein reactivity.**

To assess aqueous stability of NAP-AG, NAP-AG was added to 0.1 M phosphate buffer pH 7.4 at a final concentration of 10 μM and incubated at 37°C. At desired timepoints a 50 μl aliquot was taken and added to 150 μl LC-MS loading buffer (which is acidified to prevent AG degradation during sample analysis) containing 0.5 μM internal standard and analysed via LC-MS immediately.

To assess stability and protein reactivity of NAP-AG in HSA solution, 1 mM or 200 μM NAP-AG was added to 40 μM HSA solution and incubated at 37°C (NAP-AG:HSA molar ratio 50:1 and 10:1 respectively). At desired timepoints 150 μl aliquots of the incubation mixture were removed and stabilised via addition of 1% lactic acid (v/v) and stored at -80°C for processing. After thawing of samples at room temperature HSA was precipitated and non-irreversibly bound drug was liberated from HSA using solid phase extraction (SPE). The non-protein containing flow-through and wash fractions were evaporated to dryness under a constant stream of nitrogen, before reconstitution in LC-MS loading buffer containing 0.5 μM internal standard and analysis of loss of 1-β-NAP-AG and appearance of resulting NAP hydrolysate by LC-MS. HSA was eluted from the SPE column and re-frozen for subsequent processing. Non-irreversibly bound drug was washed from HSA via two repeats of acetonitrile protein-precipitation with aspirate discarded. 0.25 M NaOH was added to the HSA pellet and incubated at 80°C for 90 mins to liberate irreversibly bound drug. pH of the sample was neutralised via addition of 2 M HCl. A 20 μl aliquot of the neutralised sample was taken for protein determination. Liberated drug was separated from HSA using SPE. The drug containing fraction was evaporated to dryness under a constant stream of nitrogen, reconstituted in LC-MS loading buffer containing 0.5 μM internal standard and quantified by LC-MS.

**LC-MS analyses**

The analytical system consisted of a 1290 HPLC system (Agilent Technologies, Palo Alto, California) connected to an LTQ-Orbitrap XLmass spectrometer (Thermo Scientific, USA) via electrospray ionization interface. Chromatographic separation was carried out on an Acquity UPLC HSS T3 C18 column (2.1 × 150 mm, 1.8 μm) (Waters, Milford, MA, USA) at a flow rate of 1 mL/min. The column temperature was maintained at 50°C throughout all analyses. The solvents used for chromatographic separation were buffer A (10 mM ammonium acetate pH 5.0 containing 5 % ACN (v/v)) and buffer B (10 mM ammonium acetate pH 5.0 containing 95 % ACN (v/v)). Chromatographic separation of analytes was achieved by using an initial isocratic phase of 10 % buffer B for 1 min. A gradient elution from 10 % to 20 % buffer B was achieved over the following 2 mins, before the gradient was steepened from 20 % to 35 % buffer B over the following min. A final steeping of the gradient from 35 % to 90 % buffer B was achieved over the following 0.6 mins before the column was re-equilibrated to starting gradient over the next 0.4 mins. Chromatographic separation was controlled using Chemstation software (Version B.04.04, Agilent Technologies, Palo Alto, CA software). The instrument was operated in negative ion mode. The source parameters were as follows: spray voltage, 3 kV; sheath gas pressure 20 bar; auxiliary gas pressure 15 bar; source temperature 275°C; cone voltage -44V; tube lens -115V. Data were acquired from 160-900 Da with MS1 mode at a resolution of 30,000. All operations were controlled by Xcalibur software (version 2.1, Thermo Scientific, USA).

**Isolation of Adducted Human Serum Albumin Following Acyl Glucuronide Incubation by Size Exclusion Chromatography**

Following *in vitro* incubation of human serum albumin with S-Naproxen acyl glucuronide the human serum albumin was isolated via gravitational force using PD-10 desalting columns containing Sephadex G-25 medium (GE Healthcare). PD-10 columns were equilibrated via passing 25 ml of 0.1 M phosphate buffer (pH 7.4) through the column. After column equilibration, HSA samples were administered to the columns with the full sample volume allowed to reach the column before continuous addition of 0.1 M phosphate buffer (pH 7.4). Resulting eluate was collected in 500 μl fractions. Fractions were assessed for protein concentration using the Bradford assay (Bradford, 1976), and unbound drug NAP or NAP-AG via absorbance using a cuvette assay and spectrophotometer set to measure optical density at 254 nm. No co-elution of unbound drug and human serum albumin was detected. For *in vitro* T-cell experiments human serum albumin containing fractions were pooled and protein concentration determined using the Bradford assay.

## ***In vitro* diagnostics**

PBMC from study patients (1 x 10^5^/well) were incubated with NAP, DM-NAP, NAP-AG (all 50 – 600 µM) NAP-AG-albumin adduct (1mg / mL) or tetanus toxoid (1 µg/mL) / PHA (1 µg/mL) [as positive controls] in cell culture medium for 5 days. Due to concerns over NAP-AG instability compound dilutions were freshly prepared and immediately added to the cell assay. Furthermore, in separate experiments using patient PBMC and NAP-AG, 50% of medium was replenished with fresh NAP-AG containing medium every 8 hours. [^3^H]-Thymidine was added for an additional 16 hours and T-cell proliferation was measured using scintillation counting. A positive response is defined as an SI of 1.5 or above at 2 or more consecutive concentrations.

Secretion of IFN-γ and IL-22 was visualised using ELIspot by culturing PBMC (5 x1 0^5^/well) in cell culture medium in the presence of NAP or its metabolites for 48 hours. ELIspot plates were developed according to the manufacturer’s instructions and counted using an AID ELIspot reader. Fifty spot forming units or more in drug-treated wells, per million cells, above medium controls are considered positive.

## **Generation of drug-responsive T-cell clones (TCC)**

Drug-responsive T cell lines were generated via the culture of patient PBMC with NAP, DM-NAP and NAP-AG (50-400 µM) for a period of 14-28 days. Interleukin (IL)‑2 (100 U/mL) was added on days 6, 9, 20 and 25 to maintain cellular proliferation. T cell lines were restimulated with irradiated autologous PBMC’s and the respective drugs on day 14. On day 14 or 28, T cells were cloned via means of serial dilution and repetitive mitogen stimulation over a course of 4-8 weeks. Drug specificity was assessed by culturing TCC (5 x 10^4^/ 50 µL) with autologous EBV-transformed B cells (1 x 10^4^ / 50 µL) in the presence and absence of NAP, DM-NAP or NAP-AG for 48 hours (37^°^C; 5% CO_2_). [^3^H]-thymidine (0.5 µCi) was added for a further 16 hours and cellular proliferation was measured via scintillation counting. TCC which yielded a scintillation index of 2 or greater were subjected to mitogen expansion in the presence of IL-2 for a period of 14 days prior to assays to determine phenotype and functionality.

## **Phenotype and quantification of cytokine release from drug-responsive TCC**

Flow cytometry was used to characterize the phenotype of the drug-responsive TCC. First, TCC (5 x 10^4^ /50 µL) were stained with CD4-fluorescein isothiocyanate / allophycocyanin (FITC/APC) (3 µL) and CD8- Phycoerythrin (PE) (1.5 µL) and incubated at 4°C for 20 minutes prior to being analyzed using a FACS Canto II flow cytometer. A total of 10,000 events were acquired for each sample. The expression of T-cell receptor (TCR)-Vβs was analyzed on TCC (5 x 10^4^ / 50 µL) by staining using an 8-tube panel, containing 24 monoclonal antibodies, known to bind specific TCR-Vβ subtypes (IO Test Beta Mark TCR Vβ Repertoire Kit; Beckman Coulter, Brea, CA, USA). Chemokine receptor surface expression was analyzed using the following antibodies; CCR5-PE, CCR4-PE, CCR8-PE, CXCR6-PE, E-cad-PE, CXCR3-APC, CXCR2-APC, CCR9‑APC, CCR6-APC, CTLA4, CCR5-FITC, CLA-FITC, CD69-FITC, CCR1-FITC, CXCR5-FITC. Approximately 5 x 10^4^ TCC were stained and receptor expression were quantified using a FACS Canto II flow cytometer.

The cytokine secretion from drug-treated TCC was assessed using ELIspot assay. Briefly, ELIspot plates were coated overnight for cytokines: IFN-γ, IL-13, IL-17 and IL-22 (and in some instances IL-5, granzyme B, perforin and FasL). Plates were then blocked and TCC (5 x 10^4^ /50 µL) were incubated with autologous EBV-transformed B cells (1 x 10^4^ /50 µL) NAP and/or NAP metabolites at 37°C, 5% CO_2_. After a 48-hour incubation, plates were developed according to the manufacturer’s instructions and spots were counted using an AID ELIspot reader.

## **Assessment of the functionality of drug-responsive TCC**

Drug cross-reactivity was assessed by incubation of the TCC (5x10^4^ /50 µL) with autologous EBV-transformed B cells (1 x 10^4^ /50 µL) in the presence of NAP, DM-NAP, NAP-AG, alongside acetaminophen, IBU, diclofenac, aspirin (25 – 100 µM; all closely-related structures to NAP; concentration range selected from previous studies) for 48 hours (37°C, 5% CO_2_) and T-cell activity was quantified using IL-22 ELIspot as previously described.

To investigate the pathways of drug presentation to T-cells, autologous EBV-transformed B cells were pulsed for 1 or 16 hours with DM-NAP (400 µM), prior to washing 3 times to remove the unbound drug. Autologous EBV-transformed B cells were then irradiated and incubated (1 x 10^4^ / 50 µL) with drug-specific TCC (5 x 10^4^ / 50 µL) in the absence of soluble drug for 48 hours. T cell activation was quantified via analysis of proliferation or IL-22 secretion using ELISpot. Mock-pulsed EBV-transformed B cells were used as a negative control, while TCC incubated with unpulsed EBV-transformed B cells in the presence of soluble drug was used as a positive control. Secondly, EBV-transformed B cells were omitted from the assays to assess T cells proliferation or IL-22 release. Thirdly, EBV-transformed B cells were fixed with glutaraldehyde, to prevent antigen processing. TCC were then incubated with irradiated or fixed EBV-transformed B cells and soluble DM-NAP for 48 hours (37°C, 5% CO2) prior to assessment or proliferation or IL-22 release. HLA restriction was assessed by culturing TCC, EBV-transformed B cells and DM-NAP in the presence of anti-human HLA class I (Human Leukocyte antigen [HLA]‑A, B and C), HLA class II (HLA-DP, DQ and DR) (BD Pharmingen, San Jose) antibodies. T-cell proliferative responses and IL-22 secretion were measured after 48 hours. In certain experiments, the protocol was repeated with individual HLA class II allele blocking (HLA-DP, DQ and DR) antibodies.

## **Statistical analyses**

To confirm the precision and accuracy of the assays to monitor NAP-AG loss, quality control samples of 0.01, 2 and 4μM NAP-AG in 0.1M phosphate buffer or in 20μM HSA solution (all n=3) were prepared for each analytical assay and subjected to the same mass-spectrometric preparation procedure. In all data presented in this work, the accuracy and precision (coefficient of variance) of resulting read-outs from these samples was 100 ± 15 % at all points tested, excepting the lower limit of quantification of 0.01 μM which was within 100 ± 20 %.
