# Supplementary material for: Patients with naproxen‐induced liver injury display T‐cell memory responses toward an oxidative (S)‐O‐desmethyl naproxen metabolite but not the acyl glucuronide
Source: Allergy. 2023 Jul 29;79(1):200–14. doi: 10.1111/all.15830 (PMC10952231; doi:10.1111/all.15830)
Supplement: Supplementary file 2 — Figure S1. [file ALL-79-200-s001.docx]

**Supplementary Figure 1. Degradation of NAP-AG in 0.1 M potassium phosphate buffer (pH 7.4) at 37°C.** Fitted regression curve presenting the degradation of NAP-AG during incubation in 0.1 M potassium phosphate buffer (pH 7.4) at 37°C. All incubations were performed in triplicate. Data are presented as means (± standard deviation; n=3) of % (S)-NAG remaining as quantified from initial amount of NAP-AG (10 μM) that was added to the incubation mixture at t = 0 mins. Data points are fitted with exponential decay equation C=C_0_exp^(-kdeg*time)^ with parameters described in Supplemental Table 1,

**Supplementary Figure 2: Lymphocyte transformation test using DILI P2 PBMC.** Lymphocytes from NAP DILI P2 were incubated with NAP, DM-NAP or NAP-AG for a period of 5 days in a 96 well U-bottomed plate (37°C; 5% CO_2_). Culture medium was used as a negative control. [^3^H]thymidine was added for the final 16 h of the experiment and proliferation was assessed by scintillation counting. Bars denote mean of triplicate wells.

**Supplementary Figure 3. Activation of a single TCC with DM-NAP occurs in the absence of antigen processing.** (A) TCC (5 x 10^4^ / 50 μL) was incubated with autologous EBV-transformed B cells (1 x 10^4^ / 50 μL) pulsed with DM-NAP (400µM) for 16 h. The DM-NAP-pulsed EBV-transformed B cells were washed repeatedly to remove free compound prior to culturing with T-cells. Furthermore, TCC was incubated with DM-NAP (400µM) in the presence of gluturaldehyde-fixed autologous EBV-transformed B cells. Soluble drug was used as a positive control. Activation of the TCC was measured via [^3^H]thymidine.
