# Supplementary material for: Patients with naproxen‐induced liver injury display T‐cell memory responses toward an oxidative (S)‐O‐desmethyl naproxen metabolite but not the acyl glucuronide
Source: Allergy. 2023 Jul 29;79(1):200–14. doi: 10.1111/all.15830 (PMC10952231; doi:10.1111/all.15830)
Supplement: Supplementary file 3 — Table S1. [file ALL-79-200-s003.docx]

**Supplemental Table 1. Half-lives of degradation and parameters for the fitted degradation curves shown in Supplemental Figure 1 and Figure 2B.** Data are fitted to the first-order degradation rate equation C=C_0_exp^(-kdeg*time)^

| **Matrix** | **t ½ (hour)** | **t ½ (min)** | **Kdeg (min-1)** | **r2** |
| --- | --- | --- | --- | --- |
| Phosphate Buffer | 2.72 | 163.30 | 0.0042 | 0.9905 |
| 1mM NAG in 20μM HSA | 1.60 | 96.45 | 0.0071 | 0.9859 |
| 200μΜ NAG in 20μΜ HSA | 0.97 | 58.34 | 0.0118 | 0.9951 |
